# Negative Magnetoresistivity in Highly Doped n-Type GaN

**DOI:** 10.3390/ma15207069

**Published:** 2022-10-11

**Authors:** Leszek Konczewicz, Malgorzata Iwinska, Elzbieta Litwin-Staszewska, Marcin Zajac, Henryk Turski, Michal Bockowski, Dario Schiavon, Mikołaj Chlipała, Sandrine Juillaguet, Sylvie Contreras

**Affiliations:** 1 Institute of High Pressure Physics, Polish Academy of Sciences, 01-142 Warsaw, Poland; 2 Laboratoire Charles Coulomb (L2C), University of Montpellier, CNRS, 34-095 Montpellier, France

**Keywords:** GaN, magnetoresistivity, weak localization, phasing coherence time

## Abstract

This paper presents low-temperature measurements of magnetoresistivity in heavily doped n-type GaN grown by basic GaN growth technologies: molecular beam epitaxy, metal-organic vapor phase epitaxy, halide vapor phase epitaxy and ammonothermal. Additionally, GaN crystallized by High Nitrogen Pressure Solution method was also examined. It was found that all the samples under study exhibited negative magnetoresistivity at a low temperature (10 K < *T* < 50 K) and for some samples this effect was observed up to 100 K. This negative magnetoresistivity effect is analyzed in the frame of the weak localization phenomena in the case of three-dimensional electron gas in a highly doped semiconductor. This analysis allows for determining the phasing coherence time τ_φ_ for heavily doped n-type GaN. The obtained τ_φ_ value is proportional to *T*^−1.34^, indicating that the electron–electron interaction is the main dephasing mechanism for the free carriers.

## 1. Introduction

Nowadays, the applications of gallium nitride (GaN) and related compounds-based devices are very broad and high impurity doping via extrinsic sources is the key for its fabrication. For a long time silicon (Si) was the most common n-type GaN dopant and the growth of Si-doped GaN is largely controlled over a wide range of carrier densities from low-10^17^ to mid-10^19^ cm^−3^. However, in view of some structural problems occurring in heavily doped films, germanium (Ge) became, over time, the second competitor of n-type doping. While doping GaN to low and intermediate concentrations using Si and Ge has become routine, compensation mechanisms are activated under very high donor doping, limiting the maximum electron concentration achievable with either dopant. The metal-insulator transition in GaN occurs at the critical concentration of uncompensated donors equal to approximately 1.6 × 10^18^ cm^−3^ [1]. So, n-type GaN material, with free carrier concentrations higher than this critical value can be considered as a disordered system because of the high concentration of dopant atoms randomly distributed in the lattice.

To properly describe the transport phenomena in a disordered system, some quantum corrections are necessary. Within the framework of the semi classical Drude–Boltzmann model for the electrical conductivity, the quantum mechanical scattering of carriers at individual impurities is considered, but the coherent motion between scattering events and multiple scattering are neglected. However, the developing of scattering theories in which phase-coherent multiple scattering was systematically taken into account, showed that it yields a quantum correction to the conductance [2]. Electrons in a disordered sample may be scattered in either direction and the quantum correction arises when the electron travels along a closed diffusive trajectory. This phenomenon called “weak localization” enhances the resistance above the classical Drude value [3]. If one considers the case of wave functions that are scattered by the same centers (defects or impurities) but propagate in opposite directions along the same closed trajectory and, therefore, return to the origin with equal phases (systems with time reversal symmetry), then due to the coherence of both wave functions the quantum mechanical return probability is enhanced by a factor of two compared to the classical contribution to the return probability. In this sense one can talk about enhanced backscattering, carrier weak localization, as a result of quantum interference. As this additional scattering intensity exists only in the backscattering direction, it leads to an increase in the resistance of the material and lasts as long as the coherence of the scattered wave is not destroyed. The destruction of this interference is usually related to processes of electron inelastic scattering which are taken into account by electron–electron and electron–phonon interactions. The spin effect as spin-orbit coupling or spin-flip scattering by magnetic impurities also destroys the phase coherence.

However, a special role in breaking the wave function coherency is related to the magnetic field [4]. In the presence of vector potential caused by the magnetic field, the two waves propagating in the opposite directions acquire a phase difference 2eΦ/c, where Φ is the magnetic flux through the area enclosed by the electron trajectory. This phase difference breaks the constructive interference and restores the conductivity to the value it would have without the quantum interference corrections. This is observed as an increase in conductivity with magnetic field, the effect called as negative magnetoresistivity (nMR). Thus, in principle, the low-temperature electron–electron and electron–phonon interactions can be extracted from weak-localization measurements under magnetic field, which therefore represent a powerful tool for studying relaxation mechanisms of conduction electrons which is the focus of this paper.

In the case of semiconductors, while at the beginning of the development of theoretical models of localization, the research concerned low temperature effects (below 10 K) in three-dimensional (3D) systems [5,6,7,8,9,10,11,12], over time, most experimental measurements reported in the literature were focused on low-dimensional structures such as semiconductor inversion layers, heterostructures with two-dimensional electron gas, or one-dimensional semiconductor wires and their corresponding, specific theoretical models. It resulted from the interest in new structures but also from the fact that the 3D case required a large disorder to obtain localization and still quantum-interference effects are comparatively small and much less pronounced compared with those in lower dimensions [13]. Concerning the nitride semiconductors, according to the authors’ knowledge, the nMR effect was observed up to 40 K in a series of InN films and it has been established that the electron-phonon scattering is the dominant inelastic process in this 3D material [14]. The experiments with GaN compound semiconductor are reported in only two papers, both referring to two specific cases. In the work by Jastrzebski et al., which deals with GaN heavily doped with ferromagnetic dopants (Mn and Fe), only the existence of the nMR effect was signaled [15]. Attempts at a qualitative description of the phenomenon were made by Jain et al., who, however, did not describe bulk GaN, but a GaN nanowall network [16]. In the presented paper, we investigate the electrical transport properties of highly doped n-type GaN thick layers or bulk crystals with carrier Hall concentration *n_H_* ≥ 8 × 10^17^ cm^−3^. The study was carried out for materials obtained with the use of five different crystal growth technologies and doped with different donor dopants. It was found that all the samples under study exhibit nMR at a low temperature (10 K < *T* < 50 K) and for some of them this effect could be observed up to unexpected high temperatures (up to 100 K). For the first time, the free carriers scattering mechanisms in GaN were analyzed by applying the weak-localization theory. An understanding of the conductivity processes in a highly doped material is essential for device development.

## 2. Experimental Methods and Room Temperature Characterization

The list of the investigated samples and their room temperature parameters are presented in Table 1. The samples under study were prepared from n-type GaN grown using four basic techniques: metal-organic vapor phase epitaxy (MOVPE) [17], plasma-assisted molecular beam epitaxy (PAMBE) [18], halide vapor phase epitaxy (HVPE) [19], and ammonothermal growth (Am) [20]. Additionally, GaN crystallized by the High Nitrogen Pressure Solution method in a multi-feed–seed configuration (MFS) without intentional doping was also examined [21].

**Figure 1:**
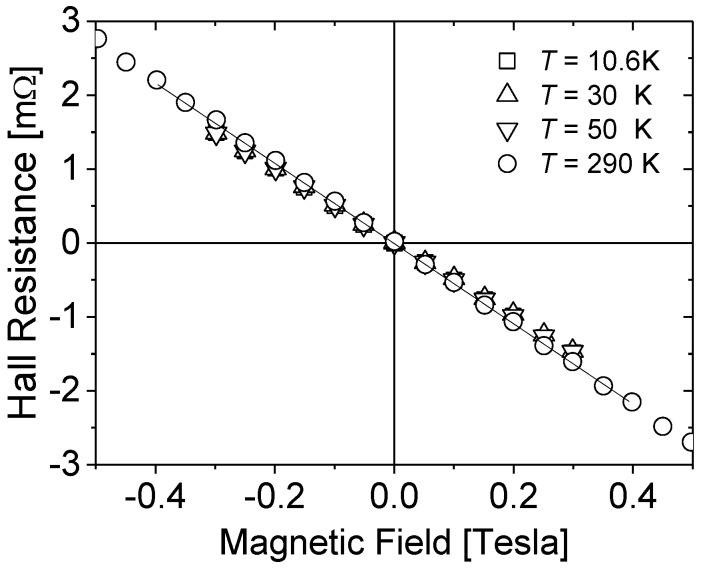
The Hall resistance *R = V_H_*/*I_s_* vs. magnetic field *B* at *T* = 10.6 K, 30 K, 50 K and at room temperature (*T* = 290 K). Results for sample H1.

In the case of MOVPE samples (indexed with letter M), only the germanium donor was applied. The layers grown by PAMBE (letter P) and HVPE (letter H) were doped with germanium or silicon. For MFS (letter F) and Am (letter A) samples the n-type conductivity was obtained by non-intentional doping with oxygen (as revealed by SIMS analysis [21,22]). For those samples that were also investigated in paper [23], their designation in both works is the same. The details of different technological procedures applied to growth of GaN materials under study are described in paper [23]. The samples were cut into 5 mm × 5 mm squares and electrical contacts were placed in the corners of each sample. Ohmic contacts were formed by the evaporation of Ti (200 nm)/Au (700 nm) electrodes and subsequent annealing in N_2_ ambient at 1000 K. For low-temperature measurements a He-free cryostat enabling measurements at temperatures ranging from 10 K was used. The temperature was stabilized with a precision better than 0.1 K. The resistivity measurements were performed using the van der Pauw method taking the average of all current configurations. Likewise, the van der Pauw approach [24] was used for the Hall Effect experiments. During all the electrical measurements, the current I_s_ through the sample was kept adequately low to ensure ohmic conditions. As an example of typical behavior, Figure 1 presents the results of the Hall Effect (Hall voltage *V_H_*) measurements as a function of magnetic field at different temperatures for sample H1. The Hall resistance *R* = *V_H_*/*I_s_* vs. *B* dependences at *T* = 10.6 K, *T* = 30 K, *T* = 50 K and at room temperature (*T* = 290 K) are practically identical. This dependence on temperature is characteristic for highly doped material [23] and indicated that for the all samples under study; the conduction process, in the whole investigated temperature range, is related to only one type of carriers, i.e., the free electrons in the conduction band.

## 3. Magnetoresistivity as a Function of Temperature

**Figure 2:**
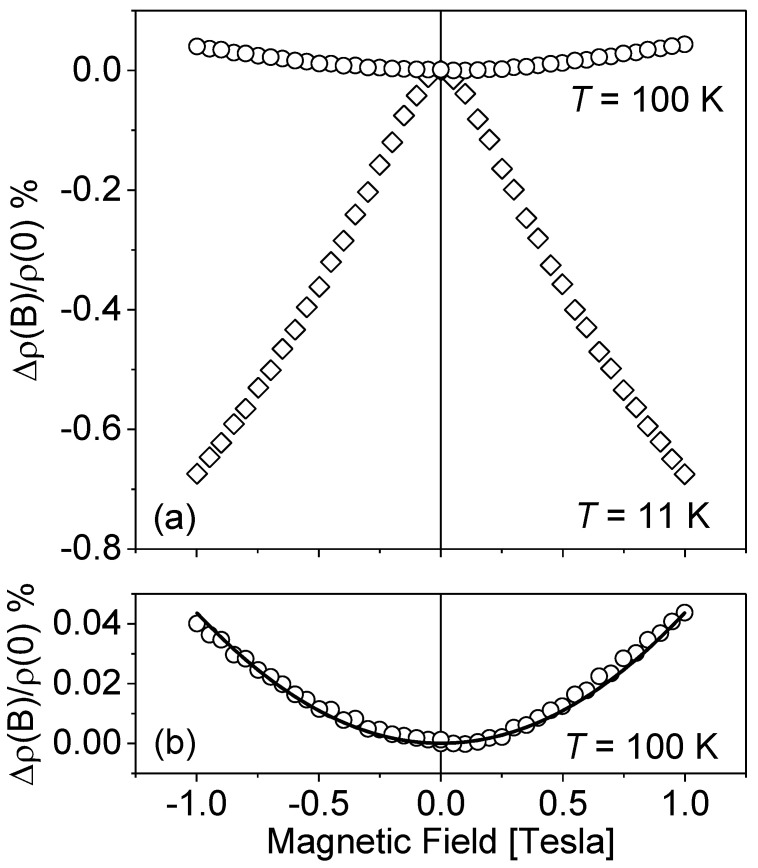
Relative variation of resistivity Δ*ρ*(*B*)/*ρ*(0) vs. magnetic field *B*. (**a**) Results for sample M5, respectively, at *T* = 100 K (circles) and *T* = 11 K (diamonds). (**b**) Zoom on 100 K results. Solid line: Gauss model Δ*ρ*(*B*)/*ρ*(0) = *β* × *B*² with the fitting parameter *β* = 4.1 × 10^−4^ (*T*^−^²).

A typical character of resistivity *ρ* on the magnetic field dependence is presented in Figure 2, where the results of *ρ*(*B*) measurements for sample M5 are plotted at *T* = 11 K and 100 K.

The positive magnetoresistivity effect observed at higher temperatures (*T* = 100 K) can be analyzed in the frame of classic electron transport phenomena and is given by the following equation [25]:

Δ*ρ*(*B*)/*ρ*(0) = *Aµ*²*B*² (1)

where Δ*ρ*(*B*) = *ρ*(*B*) − *ρ*(*B* = 0), *µ* is the carrier mobility and *A* is the scattering factor related the relaxation time.

The fitting procedure gave the value *Aµ*²= 4.1 × 10^−4^ (*T*^−2^). Taking into account the mobility of sample M5 at *T* = 100 K equal to 175 cm²/Vs, the fitting result corresponds to *A* = 1.35, which is an expected value between an ionized impurity scattering mode and acoustic phonon modes.

In the low temperatures range, the opposite effect of decreasing resistance as a function of the magnetic field is observed. This is assumed to be related to the phenomenon of weak localization and its dependence on temperature is investigated in the following part of the paper. To study this effect, the data should be analyzed using Kawabata’s equations [26] for conductivity corrections with a magnetic field in a 3D system. At low temperature, one has to distinguish between two different scattering times of the conduction electrons: elastic scattering time *τ_o_* and inelastic scattering time *τ_φ_* (phase coherence time after which the coherent backscattering disappears). Using these two parameters, Kawabata derived an expression for the variation of the conductivity as a function of magnetic field in the absence of spin-orbit and magnetic interactions. Depending on the parameter *δ* defined as:

(2) $$\delta =\frac{3l^{2}}{4\lambda \lambda_{\varepsilon }}$$

with *l* being the cyclotron length and *λ*, *λ_ε_* elastic and inelastic mean free path, respectively, he proposed two possible types of behavior of Δ*σ*(*B*,*T*) [10]:

If *δ* << 1: Δ*σ* is independent of the parameters characterizing the system and can be expressed by the universal formula:

(3) $$\Delta \sigma \left(B,T\right)=0.605\cdot \left(\frac{e^{2}}{2\pi^{2}\hbar }\right){\left(\frac{eB}{\hbar }\right)}^{1/2}$$

If *δ* >> 1: Δ*σ* it is done by a parabolic dependence on *B*:

(4) $$\Delta \sigma \left(B,T\right)=\left[\frac{\sigma_{o}}{12\sqrt{3}}\right]{\left[\frac{\tau_{\varphi }}{\tau_{o}}\right]}^{3/2}{\left[\frac{e\tau_{o}}{m^{*}}\right]}^{2}B^{2}$$

**Figure 3:**
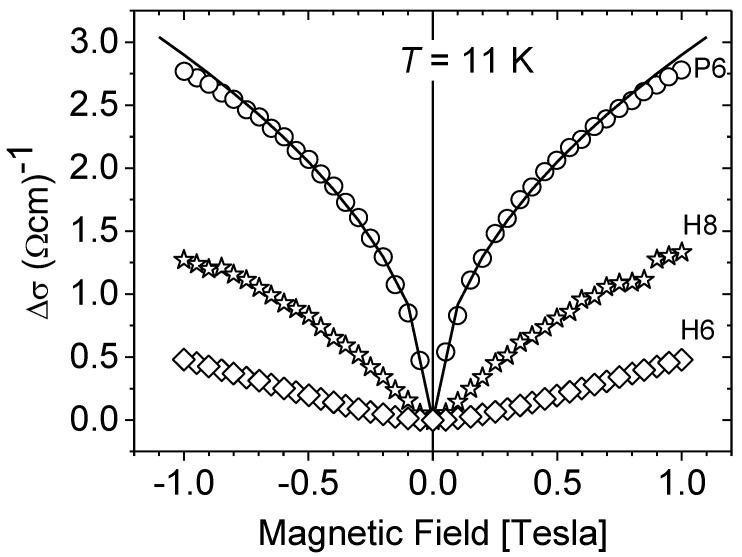
Δ*σ* vs. magnetic field *B*. Results at *T* = 11 K for samples with different, increasing carrier concentration: -*n_H_* = 3.1 × 10^18^ cm^−3^ (diamonds, sample H6); -*n_H_* = 1.1 × 10^19^ cm^−^^3^ (stars, sample H8); -*n_H_* = 1.2 × 10^20^ cm^−^^3^ (circles, sample P6). Solid line—Kawabata’s universal Equation (3).

In the above equations, *σ_o_* is the conductivity in the absence of magnetic field, *e* is the electron charge, m*—the carrier effective mass (here m* = 0.22 *m_o_*), and ℏ, *π* have their usual meanings. In the case where Equation (4) may be applied, the experimental results can be analyzed treating the phasing coherence time τ_φ_ as the fitting parameter. The experimentally observed low-temperature changes in the conductivity with the magnetic field Δ*σ*(*B*) = *σ*(*B*) − *σ_o_* for different carrier concentrations are presented in Figure 3. Although the investigated nMR effect is weak (in the best case it does not exceed 1%), the observed trends do not raise any doubts. As can be seen from the figure, the criteria that allow for applying Equations (3) or (4) to describe the conductivity corrections Δ*σ*(*B*) strongly depend on the carrier concentration in the sample.

In the case of *n_H_* > 1 × 10^20^ cm^−3^ (sample P6), the experimental dependence Δ*σ*(*B*) is perfectly described by the universal Equation (3) with the magnetic field dependence~√*B*. However, as the carrier concentration is lowered, this dependence changes its character so that for *n_H_*~1 × 10^19^ cm^−3^ the Δ*σ*(*B*) dependence becomes similar to a parabolic dependence (Δ*σ*~*B*²) and consequently can be analyzed under Equation (4).

**Figure 4:**
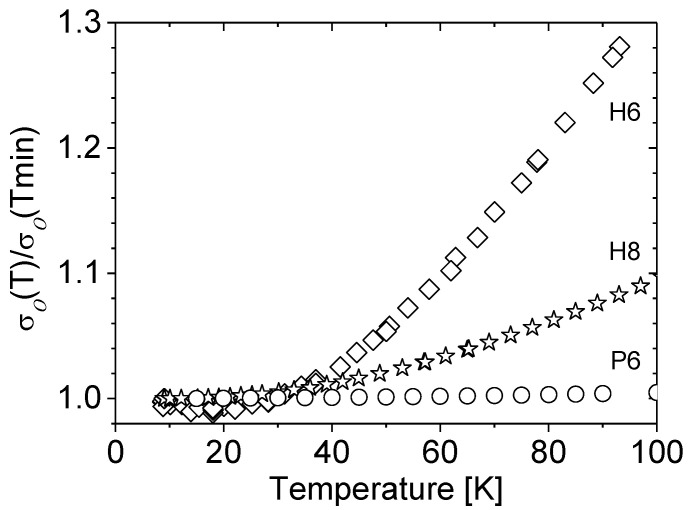
Relative variation of conductivity *σ_o_*(*T*)/*σ_o_*(*T*_min_) vs. temperature. Results for samples with different, increasing carrier concentrations: -*n_H_* = 3.1 × 10^18^ cm^−3^ (diamonds, sample H6); -*n_H_* = 1.1 × 10^19^ cm^−^^3^ (stars, sample H8); -*n_H_* = 1.2 × 10^20^ cm^−^^3^ (circles, sample P6).

Figure 4 shows, for the discussed three samples, relative changes in their conductivity with temperature. Up to approximately 40 K, the conductivity of all the samples is practically independent of the temperature, while an increase in the conductivity with temperature observed above 40 K appears only for some samples.

**Figure 5:**
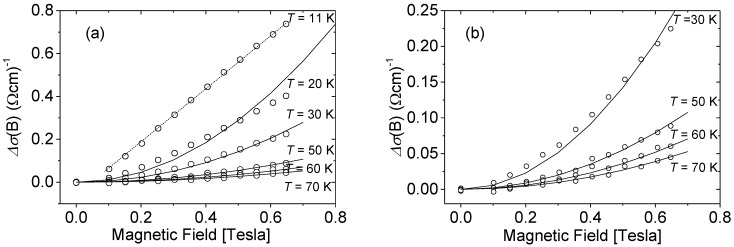
Δ*σ* vs. magnetic field *B* at different temperature. Results for sample M4 with *n_H_* = 6.9 × 10^18^ cm^−3^. Solid lines—fitting procedure. (**a**): complete set of results. For *T* < 30 K, the parabolic equation for Δ*σ*(*B*) dependency cannot be fitted. (**b**): The fitting procedure can be applied to the high-temperature part of the results with the fitting parameter τ_φ_ equal to: *T* = 30 K: *τ_φ_* = 1 × 10^−12^ s; *T* = 50 K: *τ_φ_* = 5.3 × 10^−13^ s; *T* = 60 K: *τ_φ_* = 4 × 10^−13^ s; *T* = 70 K: *τ_φ_* = 3.3 × 10^−13^ s.

The temperature dependence becomes stronger as the concentration of carriers in the sample decreases. As a consequence of the tendency shown in Figure 3, even if the nMR effect was observed in all samples under study, only for some of them was it possible to evaluate the phasing coherence time τ_φ_. For these samples, the magnetoresistance data vs. magnetic field were fitted using Kawabata’s Equation (4), treating the phasing coherence time as the fitting parameter. As an example, Δ*σ*(*B*) for sample M4 with carrier concentration *n_H_* = 6.9 × 10^19^ cm^−3^ is presented in Figure 5. While the results at low temperature do not allow for the fitting of the parabolic dependence of Δ*σ*(*B*) (Equation (4)), this condition is met by increasing the temperature.

**Figure 6:**
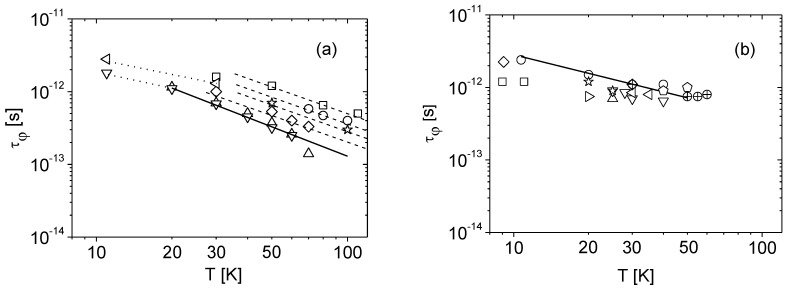
The phasing coherence time *τ_φ_* derived from the nMR data versus temperature for different samples. (**a**): MOVPE samples: ∇ M2; Δ M3; ◊ M4; PAMBE samples: ☆ P6; ○ P15; HVPE samples: ◁ H1; □ H3; The data points are given with dashed and dot parallel lines as a guide to the eye. Solid line, fitting for ∇ and Δ samples: *τ_φ_*(*T*) = 6.2 × 10^−11^ × *T*^−1.34^ (s). (**b**): Am samples: ○ A1; ◊ A2; □ A4; Δ A5; ∇ A6; ◁ A7; 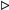 A8; MFS samples: Ψ F1; 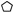 F2; ⊕F3; ◊ 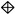 F4; Solid line: *τ_φ_*(*T*) = 2 × 10^−11^ × *T*^−0.85^ (s).

The phasing coherence time dependence on temperature τ_φ_(*T*) determined this way for several samples are presented in Figure 6. In Figure 6a are presented the results for MOVPE, PAMBE, and HVPE samples. In the temperature range *T* > 30 K, the best power-law fit of this set of data is of the form:

*τ_φ_*(*T*) = G × 10^−11^ × *T*^−1.34^ (s)

where G value is characteristic of each sample.

For *T* < 30 K, the results for samples H1 and M2 suggest a much weaker dependence on temperature *τ_φ_*(*T*)∝ *T*^−0.85^. The results for Am and MFS samples are presented in Figure 6b. For these samples the phasing coherence time *τ_φ_* could be determined at a lower temperature range *T* ≤ 40 K and its temperature dependence can be described by:

*τ_φ_*(*T*) = 2 × 10^−11^ × *T*^−0.85^ (s),

with the exponential term consistent with the results in Figure 6a for the same, lower temperature range.

It should be noted that in the case of the of Si dopant, the phasing coherence time τ_φ_ was determined only for one sample (P15) and in the highest temperature range *T* > 60 K. This may indicate that the surrounding of the Si dopant is different than in the case of Ge or O doping.

The power-law character of temperature variations of the inelastic scattering time *τ_φ_*(*T*) ∝ *T*^–^*^p^* allows for identifying the scattering mechanism dominating in the sample. The electron-phonon scattering τ_e-ph_ and the electron-electron scattering *τ_e_*_-e_ are the two main mechanisms responsible for phase-breaking [13]. In the case of the electron–phonon interaction, the standard theoretical concept scales *τ_e_*_-ph_ with temperature as *T*^−3^. In the presence of strong impurity scattering, however, the situation is less straightforward and different values of the exponent of temperature *p*, ranging from *p* = 2 to *p* = 4, have been predicted. In particular, the *T*^−2^ dependence of the relaxation rate is widely observed in experiments [13]. As can be seen in Figure 6, the best power-law fit of the experimental results correspond to *τ_φ_* ∝ *T*^−1.34^. This value of the temperature exponent is significantly lower than (*p* ≈ 2÷4) established for the electron–phonon interaction. However, this exponent value is close to *τ_e_*_-e_ ∝ *T*^−3/2^, the formula for electron–electron interaction in the case of heavily doped material. This scattering mechanism could also explain the observed decrease of the exponent p as the temperature decreases below 30 K. In this temperature range, the phasing coherence time τ_φ_ could only be measured in the less doped samples, and according to paper [27] for 3D systems approaching a metal-insulator transition a weaker temperature dependency *τ_φ_* ∝ *T*^−1^ can be expected. A similar effect of decreasing of the exponential term with decreasing temperature was also observed for the GaN nanowall network [16].

However, one should also consider the possibility that this effect is related to the experimentally observed effect at a low temperature ‘saturation’ of the electron dephasing rate [9]. This is due to the fact that the electron dephasing time *τ_φ_* can be written as:

1/*τ_φ_* =D +1/*τ_i_* (5)

where D is presumed to be constant, independent of temperature, and *τ_i_* is the relevant inelastic electron scattering time in question. As is evident from this equation, in the higher temperature range (1/*τ_i_* > D) the temperature dependence of τ_φ_ is controlled by the temperature dependence of *τ_i_*, while D determines the saturated value of the dephasing time in the limit of very low temperatures. This effect can be related to the inhomogeneities in the samples, which may be of particular importance near the Mott transition.

## 4. Conclusions

In this paper, the results of low temperature magnetoresistivity study in n type, heavily doped GaN grown by PAMBE, HVPE, MOVPE, Am and MFS were presented.

In this study, we have found that all the samples, regardless of crystal growth technique or dopant, exhibit negative magnetoresistivity (nMR, Δ*σ* > 0). For a great part of the investigated samples, it was possible to determine the temperature range where the Δ*σ*(*B*) dependency was approaching the parabolic Δ*σ* ∝ *B*² and could be analyzed in the frame of the weak localization phenomena model. As a result, for the first time, the phasing coherence time *τ_φ_* was determined for 3D electron gas in GaN. The determined values are coherent with the results for other 3D semiconductor materials.

Such an extensive occurrence of the phenomenon of nMR is worth stressing. It suggests that this particular phenomenon could be applied for more systematic studies of heavily doped materials with different elements in terms of their surrounding in the lattice. For this purpose, it would be interesting to further study Si-doped GaN for which the nMR effect was observable only for the highest temperatures.

We have performed a quantitative determination of the electronic phase coherence time as a function of temperature. At temperature *T* > 30 K, the measured dephasing rate *τ_φ_* ∝ *T*^−1.34^ agrees well with that predicted by theories based on electron–electron interactions. This result is very surprising and unexpected because at such high temperatures the role of electron phonons scattering should be significant and even dominant.

In the case of less doped samples and *T* < 30 K, a weaker temperature dependency *τ_φ_* ∝ *T*^−0.85^ was observed, which can be the signature for 3D systems approaching a metal-insulator transition. To study this phenomenon in more detail, it would be advisable to extend the measurements to the lowest possible temperature range. If it turns out that by lowering the temperature, the relaxation time tends to a constant value, conclusions can be made regarding disorder in the system. Then, the nMR phenomenon could be used to estimate the quality of the material.

## Figures and Tables

**Table 1:** Sample characteristics: Hall concentration at 300 K *n_H_* (300 K) = 1/(*eR_H_*) and Hall mobility *µ_H_* = 1/(*eρ n_H_*) at 300 K and 10 K respectively.

Dopant	N°	*n_H_* cm^−3^	*µ_H_* (300 K) cm²/Vs	*µ_H_* (10 K) cm²/Vs
Ge	M1	2.0 × 10^18^	276	108
	M2	2.8 × 10^18^	230	112
	M3	4.1 × 10^18^	216	119
	M4	6.9 × 10^18^	196	131
	M5	7.3 × 10^18^	202	135
	M9	4.4 × 10^19^	153	140
	M10	1.3 × 10^20^	120	121
Ge	H1	4.1 × 10^18^	184	85
	H2	8.8 × 10^18^	192	110
	H3	1.6 × 10^19^	127	109
	H4	6.1 × 10^19^	107	103
Si	H5	1.3 × 10^18^	297	108
	H6	3.1 × 10^18^	246	80
	H7	7.9 × 10^18^	144	86
	H8	1.1 × 10^19^	153	108
Ge	P3	1.7 × 10^18^	186	33
	P6	1.2 × 10^20^	88	88
	P10	5.3 × 10^20^	47	50
	P11	7.9 × 10^20^	33	31
Si	P12	8.3 × 10^19^	140	142
	P15	2.7 × 10^19^	97	88
O	A1	5.2 × 10^18^	164	91
	A2	8.1 × 10^17^	336	9
	A3	1.4 × 10^19^	122	101
	A4	1.1 × 10^18^	249	17
	A5	2.9 × 10^19^	129	125
	A6	8.0 × 10^18^	187	140
	A7	2.0 × 10^19^	153	136
	A8	4.4 × 10^19^	123	119
O	F1	7.4 × 10^19^	77	78
	F2	5.7 × 10^19^	65	65
	F3	9.9 × 10^19^	56	55
	F4	1.2 × 10^20^	49	56

## Data Availability

Data is contained within the article.
